# Permeability of boron- and nitrogen-doped graphene nanoflakes for protium/deuterium ions†

**DOI:** 10.1039/d1ra09398c

**Published:** 2022-01-31

**Authors:** Iram Gul, Muhammad Yar, Arsalan Ahmed, Muhammad Ali Hashmi, Khurshid Ayub

**Affiliations:** Department of Chemistry, COMSATS University, Abbottabad Campus Abbottabad KPK Pakistan 22060 khurshid@cuiatd.edu.pk +92-992-383441 +92-992-383591-6; Department of Chemistry, Division of Science & Technology, University of Education 54770 Lahore Pakistan

## Abstract

Two-dimensional (2D) monolayer nanomaterials are the thinnest possible membranes with interesting selective permeation characteristics. Among two-dimensional materials, graphenes and hexagonal boron nitride (h-BN) are the most promising membrane materials, which can even allow the separation of proton isotopes. The current work aims at understanding the higher reported permeability of h-BN by sequential doping of B and N atoms in graphene nanoflakes. The kinetic barriers were calculated with two different models of graphenes; coronene and dodecabenzocoronene *via* zero-point energy calculations at the transition state for proton permeability. The lower barriers for h-BN are mainly due to boron atoms. The trends of kinetic barriers are B < BN < N-doped graphenes. The permeation selectivity of graphene models increases with doping. Our studies suggest that boron-doped graphene models show an energy barrier of 0.04 eV for the permeation of proton, much lower than that of the model graphene and h-BN sheet, while nitrogen-doped graphenes have a very high energy barrier up to 7.44 eV for permeation. Therefore, boron-doped graphene models are suitable candidates for proton permeation. Moreover, the presence of carbon atoms in the periphery of BN sheets has significant negative effects on the permeation of proton isotopes, an unexplored dimension of the remote neighboring effect in 2-D materials. This study illustrates the need for permeation study through other hetero-2D surfaces, where interesting hidden chemistry is still unexplored.

## Abstract 2

Two-dimensional (2D) monolayer nanomaterials are the thinnest possible membranes with interesting selective permeation characteristics.

## Graphical abstract

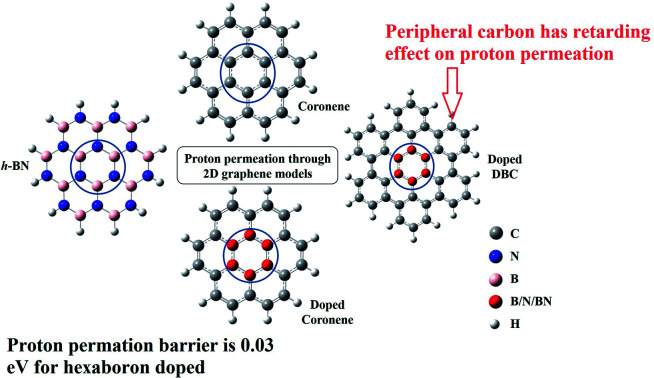

## 1 Introduction

Membranes are selectively permeable boundaries that allow the passage of certain particles through it while restricting others due to a specific driving force.^1^ These are the elemental constituents of a large variety of chemical, biological, and physical systems, and are used in almost all the things from cellular level to advanced mechanical sensing. Membranes are useful in separation processes such as water purification, waste water treatments and desalination of ocean water.^2^ Moreover, they can also be used for the purification of different gases.^3^ However, the membranes also possess a number of medical applications such as membrane oxygenation, hemodialysis, artificial kidney, artificial pancreas, artificial liver and bioreactors.^4^ Furthermore, biological membranes present in the living cells are responsible for the selective transport of ions and other useful polar molecules across the lipid bilayer.^5^ The membranes may be natural or synthetic.

Natural or biological membranes are responsible for complicated and selective transport within the living beings. These membranes are highly selective, thereby controlling the incoming and outgoing transport of all particles, maintaining the conformation of the cellular fluid.^6^ Natural membranes are very much important for the normal functioning of all the cells. They perform functions quite rapidly with less energy consumption, commonly *via* active transport.^7^ These membranes regulate the rate of important information transfer among cells either by identifying received pulse molecules from different cells, or through forwarding electrical and chemical signals to the cells.^8^ On the other side, synthetic membranes are not as complicated in their anatomy and functions as natural membranes. These membranes possess only nonresistant transport characteristics and these are mostly less selective and energy-saving. Examples of synthetic membranes include inorganic,^9^ polymeric,^10^ mixed matrix,^11^ and carbon molecular sieve membranes.^12^ In general, they contain effectively enhanced chemical and mechanical stability, principally at high temperatures. Their selectivity is analyzed through a sieve-like structure according to their capacity or through a homologous structure according to the solubility and diffusivity of solutes.^13^ Synthetic membranes play an important role in reverse osmosis, filtration processes, pervaporation, emulsion liquid membranes, solvent extraction through membranes, various membrane reactors, gas permeation and supported liquid membranes.^14^ Among the synthetic membranes, graphene membranes are found to be very attractive for scientists because of their extraordinary properties.

However, an ideal layer of graphene (non-defected) is not permeable for gas atoms (even for He atoms), which are repulsed due to the electron density of the graphene's aromatic rings.^15^ Thus, the synthetic production of nano pores by various methods such as electron beam treatment or production of feasible initiators for the self-building of graphene structures is examined.^16^ Some computational studies were performed on the formation of pores in graphene sheets for important applications such as separation of H_2_ from CH_4_, separation of helium from other noble gases and CH_4_, specific transport of ions, DNA characterization and H_2_O filtration.^17^ In all separation procedures based on the graphene and other porous membranes, sieving is a feasible process that permits the passage of particles smaller than graphene pores, but stops the transport of larger ones.^18^ For example, quantum mechanical studies explained that graphenes with a pore size of about 3.3 Å show greater specificity (high perforation rate) to hydrogen than to other gases.^19^ This specific sieving property can be generally postulated as the greater surface adsorption of bigger particles by graphene sheets, known as the preferential surface adsorption. This feature can boost the passage of particles *via* mono-layered graphene membranes.^20^ In addition to graphene monolayers, there are certain other 2D analogues of graphenes that have been reported, which include hexagonal boron nitride (h-BN),^21^ borophene,^22,23^ stanene,^24^ silicene,^25^ black phosphorus,^26^ germanene,^27^ and transition-metal dichalcogenides (*e.g.*, MoS_2_).^28^ Recently, 2D crystals of metal organic frameworks (MOFs),^29^ covalent organic frameworks (COFs),^30^ and MXenes^31^ have also been reported in the literature, and are found to exhibit remarkable properties similar to graphenes. Moreover, the one atom thickness of graphene monolayers and their analogues makes them the thinnest membranes with an extraordinary specific staining feature for the separation of gases and dissolved ions.^32,33^

These 2D single-layer nano-sized materials are utilized as the thinnest membranes with selective staining characteristics for H-isotopes.^34^ Recently, it has been reported that the monolayer of graphenes and h-BN exhibit subatomic selectivity, *i.e.*, easier permeation of protium ions (H^+^) compared to heavier deuterium ions (D^+^).^35^ Geim *et al.* discovered that these 2D single-layered membranes are able to separate proton isotopes such as protium and deuterium. They demonstrated that the dissimilarity in the transportation velocity is due to the difference in zero-point energies, which provides a comparatively high selectivity ratio for these two isotopes. The sieving energy barriers (*E*_b_) calculated experimentally for graphenes and h-BN are 0.8 eV and 0.3 eV, respectively.^36^ Furthermore, the separation factor was estimated to be about 10 for H^+^/D^+^. This factor is much larger than that of the previously reported factor of <2.5.^37^ Moreover, it has been shown that the permeation of proton through the 2D nanomaterials is thermally activated. This isotopic separation is based on velocity when transported by a single layer of graphene membranes. In addition, it has been reported in the literature that the doped graphene permits rapid permeation of protium and deuterium ions as compared to pure and normal graphenes.^34^ Separation of isotopes is of great importance for various analytical and tracing technologies, and thus, makes 2D graphenes and their analogues very promising for separating proton isotopes.

Zhang *et al.* theoretically studied the permeability of proton isotopes through graphenes and h-BN while using small coronene and its analogue models. Their results were consistent with the experimental studies that these nanomaterials are selective for protium than deuterium and tritium. Moreover, the permeability of h-BN is higher than that of graphenes. This study provides rationalization for the high permeability of h-BN than graphenes, but it does not unveil the specific effects of boron and nitrogen atoms. We became interested in studying the effect of boron and nitrogen atom doping in graphenes on the permeability for proton isotopes. In this regard, multiple boron, nitrogen and BN (co-doped) atoms are doped on the coronene model.

## 2 Computational methodology

Density functional theory (DFT)-based methods are used to study the permeation of proton isotopes (protium (H^+^), deuterium (D^+^) and tritium (T^+^)) through two-dimensional graphene membranes.^36^ All calculations were performed using the Gaussian 09 package.^38^ All results were visualized using a graphical interface such as GaussView 5.0.^39^ Frequency analysis was performed by the ωB97XD method of DFT with the 6-31++G(d,p) basis set. ω B97XD is a range separated hybrid functional.^40,41^ It was one of the first DFT methods that had a significant improvement over Hartree–Fock. H^+^, D^+^ and T^+^ have the same electronic structures. Therefore, energies of H^+^, D^+^ and T^+^ are the same. Because of this reason, these isotopes cannot be differentiated based on electronic energies. For their differentiation, zero-point correction and Gibbs free energies are calculated. In this work, two models were taken for graphenes, *i.e.* coronene and dodecabenzocoronene (DBC) and doping with boron and nitrogen was studied for proton isotope permeation. Moreover, N, B, BN, doped coronene and dodecabenzocoronene were also used to study the effect on the proton permeation rate. Furthermore, zero-point correction difference was also studied for selectivity ratios for H^+^, D^+^ and T^+^ isotopes to understand the permeability behavior in various cases.

## 3 Results and discussion

The basic purpose of this study is to find out the effect of doping on the permeability of graphene models for proton isotopes. The permeation barriers were analyzed not only to describe the selectivity for various isotopes, but also to realize the effects of various dopants. The dynamics of proton isotopes through different graphene membranes were studied under the gaseous state at 298 K temperature and 1 atm pressure.

The energy of formation per atom for boron- and nitrogen-doped systems was calculated, and is given in Table S1.† The formation energy per atom for pure coronene is −160.14 kcal mol^−1^, and it decreases gradually for each boron doping. For example, the formation energy for a mono-boron-doped system is −157.02 kcal mol^−1^ and it drops further to −153.61 kcal mol^−1^ for a bis-boron-doped coronene system. The drop in the energy of formation is also seen in a nitrogen-doped system but the decrease here is less pronounced as compared to boron-doped systems. Co-doped BN coronene systems also have low energy of formation as compared to pure coronene.

### 3.1 Permeation of proton isotopes through a pristine coronene model

**Fig. 1:**
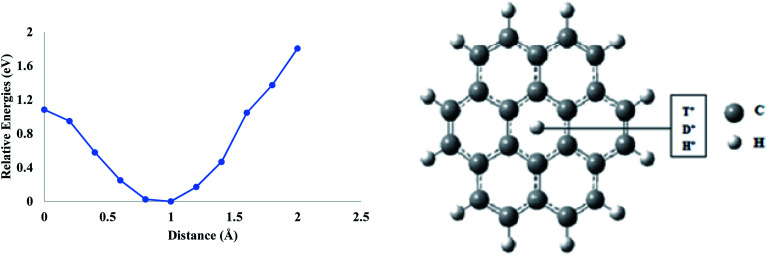
Permeation of proton isotopes through a pure coronene model.

First, a pure coronene model was taken to evaluate the permeability of proton isotopes. It is selected since it resembles the graphene fragment, and this model has been used previously in the theoretical studies. The hexagon present in the center has a pore environment very similar to the hexagonal ring in the graphene monolayer. In this study, the proton isotopes were individually placed at a vertical distance of ∼2 Å normal to the central pore and were allowed to approach the cavity. It was observed that, as the proton approaches towards the center, the total energy of the system decreases gradually up to ∼1 Å. On further translation, the total energy begins to rise, and the maximum energy is obtained when the proton is placed exactly in the center of coronene (Fig. 1). All the three isotopes have shown similar types of behavior when they are individually allowed to pass through the hexagonal ring of the coronene model. The calculated barrier (based on zero-point corrected energies) for H^+^ is 1.08 eV, which increases to 1.10 eV for D^+^ (Table 1). The highest activation barrier was calculated for T^+^ (1.14 eV). The lowest activation barrier of H^+^ indicates its high permeation rate through coronene as compared to D^+^ and T^+^. This may be attributed to the difference in ZPE between the isotopes.^35^ The lighter H^+^ possesses greater ZPE compared to the heavier isotopes and, hence, faces a lower barrier.

**Table 1:** Calculated permeation barriers (*E*_p_) for proton isotopes through boron-doped coronene sheets

Model	Permeation barriers (*E*_p_) in eV		
	Protium (H^+^)	Deuterium (D^+^)	Tritium (T^+^)
Pristine coronene	**1.08**	**1.10**	**1.14**
Pure h-BN	**0.65**	**0.66**	**0.67**
B-coronene	**0.77**	**0.79**	**0.80**
2B-coronene	**0.63**	**0.64**	**0.65**
3B-coronene	**0.46**	**0.47**	**0.48**
4B-coronene	**0.18**	**0.19**	**0.20**
5B-coronene	**0.17**	**0.18**	**0.19**
6B-coronene	**0.04**	**0.05**	**0.06**

**Fig. 2:**
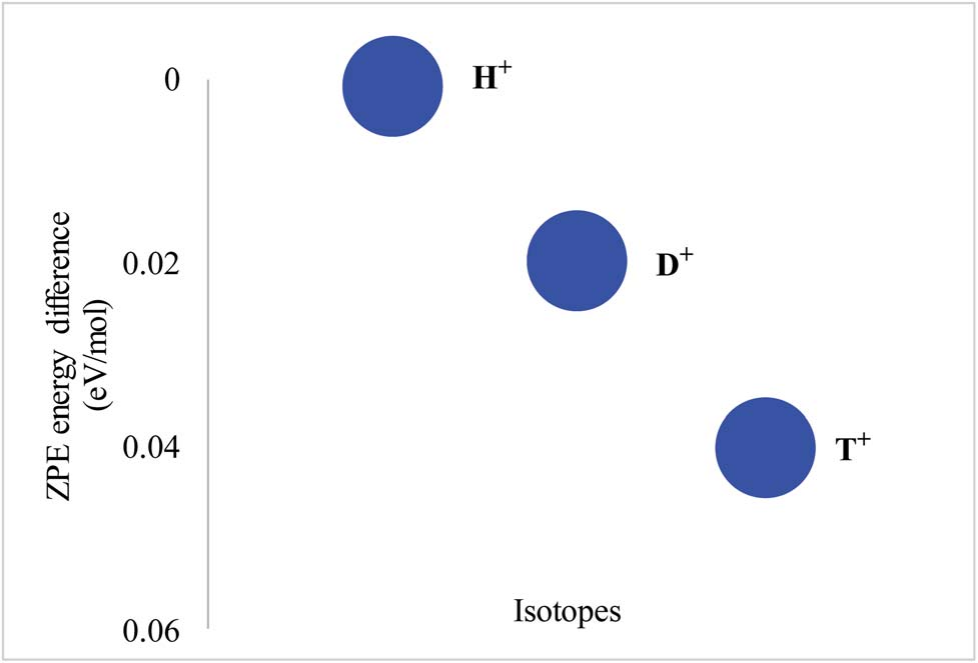
Zero-point correction differences for H^+^, D^+^ and T^+^ in the pure coronene model.

In order to verify this concept, zero-point energies for all proton isotopes were computed using the same level of theory. Zero-point correction differences were calculated by taking H^+^ as a reference, while zero-point corrected values of other two isotopes were subtracted from the reference value, *i.e. E*_*Z*PE_(H^+^)–*E*_*Z*PE_(D^+^) and *E*_*Z*PE_(H^+^)–*E*_*Z*PE_(T^+^), respectively (Fig. 2). The difference for H^+^ is 0.0 kJ mol^−1^ while for D^+^ and T^+^, the computed values are 0.0060 and 0.0087 eV mol^−1^, respectively (Table 2). Based on these ZP correction differences, the tunneling reaction constant (selectivity ratios) was calculated for isotope separation using the Arrhenius equation (K_1_/K_2_ = e^−Δ*E*_a_/RT^). The selectivity ratios K_H^+^_/K_D^+^_ and K_H^+^_/K_T^+^_ for proton isotopes are 1.27 and 1.40, respectively. The results indicate that the heavy isotopes, deuterium (D^+^) and tritium (T^+^) permeate slower than lighter protium (H^+^) isotopes through the coronene model.

**Table 2:** Zero-point energy differences (eV) and tunneling constant (selectivity ratios) of proton isotopes passing through boron-doped coronene sheets

Model	ΔZPE_(H^+^-D^+^)_	ΔZPE_(H^+^-T^+^)_	K_H^+^_/K_D^+^_	K_H^+^_/K_T^+^_
Pristine coronene	0.0060	0.0087	1.27	1.40
Pure h-BN	0.019	0.041	2.18	4.84
B-coronene	0.0074	0.0088	1.33	1.41
2B-coronene	0.0055	0.0084	1.24	1.38
3B-coronene	0.0032	0.0050	1.13	1.21
4B-coronene	**0.0036**	**0.0056**	**1.15**	**1.24**
5B-coronene	**0.0020**	**0.0034**	**1.08**	**1.14**
6B-coronene	**0.0015**	**0.0027**	**1.06**	**1.11**

### 3.2 Permeation of proton isotopes through a pure h-BN sheet

**Fig. 3:**
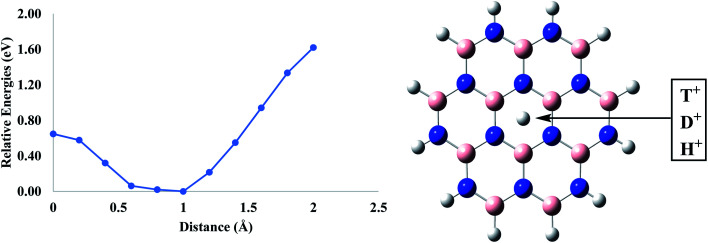
Permeation of proton isotopes through pure h-BN sheets.

Hexagonal boron nitride (h-BN) sheet is another two-dimensional nanomaterial, which contains a network of sp^2^ hybridized boron and nitrogen atoms. It has been reported experimentally by Geim and his co-workers that a monolayer of these nanomaterials favorably allows the transport of proton with a relatively lower thermal activation barrier of ∼0.3 eV.^35^ Similar to the coronene model, the h-BN fragment was taken to calculate the permeation barriers for proton isotopes. It was observed that the total energy of the system continuously decreases as the proton isotopes are approaching towards the central pore. However, from a distance of ∼1 Å, the energy begins to rise (a phenomenon similar to coronene permeation). The calculated energy barriers for H^+^, D^+^ and T^+^ are 0.65 eV, 0.66 eV and 0.67 eV, respectively (Table 1). These barriers are comparatively lower than those calculated for the pure coronene model with the energy difference of almost 0.46 eV. This barrier difference is comparable with the previously reported ones of ∼0.46–0.5 eV.^34,35^ From the previously reported results and the calculations performed, it is clear that the h-BN monolayer allows faster permeation of proton isotopes as compared to the pure graphene (Fig. 3).

Similar to the pure coronene, zero-point correction differences were also calculated for the h-BN sheet. The calculated differences for D^+^ and T^+^ were 0.019 and 0.041 eV mol^−1^ with respect to that of H^+^, respectively (Table 2). Moreover, the tunneling constant (selectivity ratios K_H^+^_/K_D^+^_ and K_H^+^_/K_T^+^_) values for D^+^ and T^+^ with respect to H^+^ are 2.18 and 4.84, respectively. These ratios are lower than those calculated for coronene. The results have indicated that the proton isotopes pass through the h-BN sheet with a greater velocity than that in case of pure graphenes, with a slightly lower selectivity.

### 3.3 Permeation of proton isotopes through boron-doped coronene models

In order to enhance the permeation properties of graphene sheets, these materials were modified by doping. Therefore, the coronene sheet was further modified by substitutional doping. An ideal dopant should not affect the planarity of coronene. The boron element is a more important dopant, which can induce new and complementary properties useful for alternative devices and technologies such as sensors and photovoltaics.^42^ The larger radius of the boron dopant as compared to that of carbon is responsible for the increase in C–B bond length.^43^ Thus, a disorder is produced in the hexagonal lattice by placing dopants at central rings.^44^

**Fig. 4:**
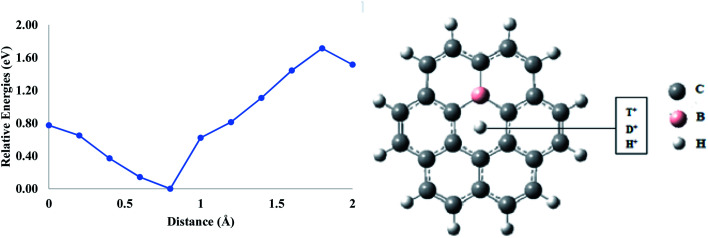
Permeation of proton isotopes through mono-boron-doped coronene model.

In the case of the mono-boron-doped coronene model, it was observed that the energy barrier for the permeation of H^+^ was reduced to 0.77 eV compared to what it was in pure coronene (1.08 eV) (Table 1). Similarly, these barriers were dropped to 0.79 and 0.80 eV for D^+^ and T^+^ permeation, respectively. These energy barriers are comparatively lower than those of pure coronene models. This is due to the p-type nature of boron that produces a hole in the coronene sheet and, therefore, increases the pore size compared to the one present in pure coronene. As a result, the permeation of proton isotopes through mono-boron-doped coronene is higher than that of pure coronene (Fig. 4).

**Fig. 5:**
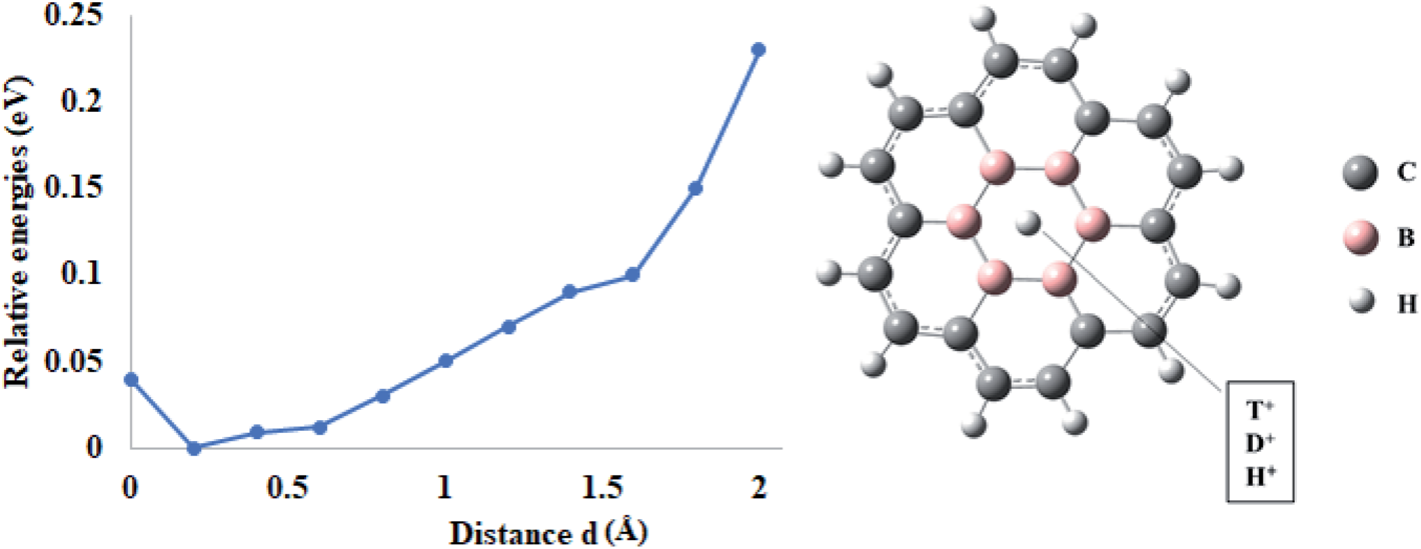
Permeation of proton isotopes through 6B-coronene.

The permeation barriers were found to be further reduced when two boron dopants were incorporated in the center of the hexagon ring. The calculated energy barrier for H^+^ is 0.63 eV. The calculated energy barriers for D^+^ and T^+^ are 0.64 and 0.65 eV, respectively (Table 1). Again, the lower barriers are due to boron atoms that are distorting the hexagonal ring and, thereby, increasing the pore size. Since two boron atoms are incorporated, the size of the ring gets even larger than what it was in the case of mono-doped coronene. Therefore, the rate of proton permeation is faster in bis-doped coronene than in mono-boron-doped coronene. A similar type of trend was observed for multiple-doped systems as well. In the case of tris-B-doped coronene, the calculated barriers for proton isotopes (H^+^, D^+^ and T^+^) are 0.46, 0.47 and 0.48 eV for H^+^, D^+^ and T^+^, respectively. When four boron atoms are incorporated in the hexagon ring, the barriers are further reduced to 0.18, 0.19 and 0.20 eV for H^+^, D^+^ and T^+^, respectively. The lowest barriers for proton permeation are observed when all the six members of the ring are replaced with boron atoms. The calculated barriers for hexa-boron-doped coronene (6B-coronene) are 0.04, 0.05 and 0.06 eV for H^+^, D^+^ and T^+^, respectively (Fig. 5).

The selectivity ratios for proton isotopes through doped coronene models were also calculated from zero-point correction differences. In case of B-coronene, it was observed that selectivity ratios K_H^+^_/K_D^+^_ and K_H^+^_/K_T^+^_ are 1.33 and 1.41, respectively (Table 2). These selectivity values are almost comparable with pure coronene model which indicate that mono-doped coronene offers lower permeation barriers than pure coronene for proton isotopes but with the same selectivity. Similarly, for 2B-coronene, the calculated selectivity ratios K_H^+^_/K_D^+^_ and K_H^+^_/K_T^+^_ are 1.24 and 1.38, respectively. However, it was observed that as the number of dopants is increasing in the hexagonal ring, the selectivity ratios for the corresponding models are decreasing for proton isotopes. The calculated selectivity ratios K_H^+^_/K_D^+^_ and K_H^+^_/K_T^+^_ are reduced to 1.13 and 1.21, respectively in case of 3B-coronene. Similarly, these ratios are further reduced to 1.15 (K_H^+^_/K_D^+^_) and 1.24 (K_H^+^_/K_T^+^_) in 4B-coronene. The lowest selectivity ratios were calculated in 6B-coronene, which are 1.06 and 1.11 for K_H^+^_/K_D^+^_ and K_H^+^_/K_T^+^_, respectively. This gradual decrease in the selectivity of doped coronene with the increase in the number of boron atoms is due to the increase in the pore size. As the number of boron atoms increase in the hexagonal ring, they offer more distortion to the ring, and hence, the size of the pore increases, which, in turn, increases the permeability. At the same time, the selectivity of the ring decreases because it will not resist that much to the heavier isotope compared to the pure coronene.

### 3.4 Permeation of proton isotopes through nitrogen-doped coronene models

The incorporation of nitrogen atoms plays an important role especially in carbon-based materials.^45^ By incorporating nitrogen atoms, three different types of configurations were obtained such as pyrrolic N, pyridinic N, and graphitic N.^45^ Nitrogen-doped graphenes have shown significant applications in the field of biosensors and bioelectronics.^46^ Moreover, nitrogen doping in the hexagon of graphene sheets acts as a strong scattering center.^47^

**Fig. 6:**
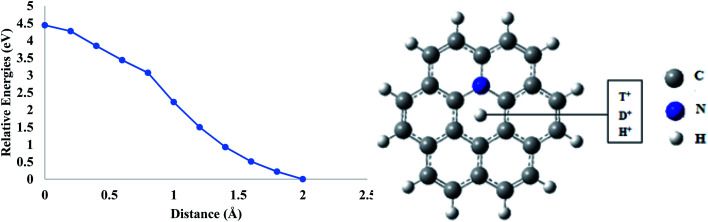
Permeation of proton isotopes through a mono-nitrogen-doped coronene model.

Similar to boron, doping of nitrogen atom in the coronene sheet has also been performed theoretically to investigate the effect of nitrogen atom on the permeability of coronene for proton isotopes. In mono-nitrogen-doped coronene model (N-coronene) (Fig. 6), when proton isotopes are individually allowed to pass through the central pore, it was observed that the total energy of the system increases continuously until the isotopes reach exactly in the center of hexagon. The energy barriers calculated for the proton isotopes are 4.44, 4.45 and 4.44 eV for H^+^, D^+^ and T^+^, respectively (Table 3). These barriers are considerably higher than those for pure and boron-doped coronene models. This is due to the high charge density of nitrogen atoms. Introduction of nitrogen atoms in the ring creates polar sites in the hexagon of the coronene model. The scattering centers in nitrogen result in the distortion of the carrier mobility^48^ which is not favorable for the permeation of proton isotopes. Therefore, the permeation of proton isotopes is slower in the case of nitrogen doping.

**Table 3:** Calculated permeation barriers (*E*_p_) for proton isotopes through nitrogen-doped coronene sheets

Model	Permeation barriers (*E*_p_) in eV		
	Protium (H^+^)	Deuterium (D^+^)	Tritium (T^+^)
N-coronene	**4.44**	**4.45**	**4.44**
2N-coronene	**4.48**	**4.47**	**4.48**
3N-coronene	**5.44**	**5.46**	**5.64**
4N-coronene	**4.96**	**4.99**	**5.02**
5N-coronene	**7.50**	**7.52**	**7.55**
6N-coronene	**7.44**	**7.46**	**7.47**

The energy barriers are slightly reduced (compared to mono-nitrogen-doped systems) when two of the nitrogen atoms are incorporated into the hexagonal ring of coronene. The calculated barrier for H^+^ and T^+^ is 4.48 eV while for D^+^, it is a bit lower, *i.e.* 4.47 eV, respectively. On further increasing the number of nitrogen atoms in the ring, an odd-even pattern of proton permeation through coronene models is observed (Table 3). For example, the proton permeation barrier through tris-nitrogen doped coronene (5.44 eV) is higher than tetra-nitrogen-doped coronene (4.96 eV). Similarly, the proton permeation barrier through penta-nitrogen-doped coronene (7.50 eV) is higher than that through the hexa-nitrogen-doped coronene (7.44 eV), although the difference is not very much pronounced. The highest barrier is observed for penta-nitrogen-doped coronene. The observed order of permeation rate is 1 < 2 < 4 < 3 < 6 < 5 (numbers represent the nitrogen atoms in the ring). This odd-even oscillation is probably due to the radical effect in nitrogen atoms, which distorts the interactions in a molecule. This distortion is not favorable for nitrogen doping, which disturbed the pattern of the permeation rate in proton isotopes.

Similar to permeation barriers, an even-odd pattern for selectivity ratios of nitrogen-doped coronene was also observed (Table 4). The highest selectivity ratios K_H^+^_/K_D^+^_ and K_H^+^_/K_T^+^_ are calculated in case of 5N-coronene (2.26 and 3.09, respectively). This is due to the obvious greatest permeation barriers for proton isotopes in 5N-coronene. As the size of the pore decreases, the permeation barriers increase and hence the pore gets more selective for the transport of lighter ions. The permeability and selectivity ratios are inversely related to one another. Any factor which increases the permeability leads to a decrease in selectivity and *vice versa*.

**Table 4:** Zero-point energy differences and selectivity ratios of proton isotopes passing through nitrogen-doped coronene sheets

Model	ΔZPE_(H^+^-D^+^)_	ΔZPE_(H^+^-T^+^)_	K_H^+^_/K_D^+^_	K_H^+^_/K_T^+^_
N-coronene	**0.0080**	**0.011**	**1.33**	**1.37**
2N-coronene	**0.010**	**0.015**	**1.49**	**1.77**
3N-coronene	**0.011**	**0.012**	**1.56**	**1.57**
4N-coronene	**0.013**	**0.017**	**1.66**	**1.94**
5N-coronene	**0.021**	**0.029**	**2.26**	**3.09**
6N-coronene	**0.009**	**0.013**	**1.42**	**1.66**

### 3.5 Permeation of proton isotopes through boron and nitrogen (BN) Co-doped coronene models

BN co-doped graphenes have been studied to investigate the structural and electronic properties of doped 2D materials.^49^ It has been reported that co doping of BN atoms can produce a variable band gap, which depends on the placement of boron and nitrogen atoms on the sheet.^50^ Such nanomaterials are very useful in opto-electronics applications.

**Fig. 7:**
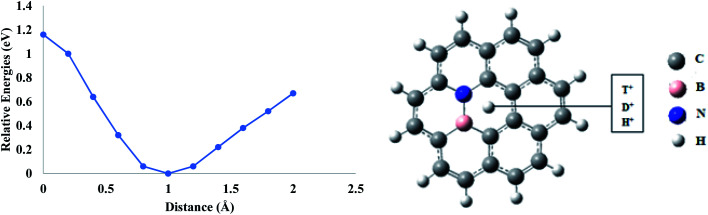
Permeation of H^+^, D^+^ and T^+^ isotopes through a BN co-doped coronene model.

In order to evaluate the effect of co-doping on the permeation of proton isotopes, boron and nitrogen atoms are doped simultaneously on the coronene sheet. First, the coronene sheet is doped by one BN fragment and the hydrogen isotopes are allowed to approach the central pore starting from the same vertical distance of 2 Å. In case of H^+^ permeation, the calculated energy barrier is 1.15 eV. Whereas energy barriers for D^+^ and T^+^ are 1.16 and 1.17 eV, respectively. These energy barriers are comparatively lower than those of nitrogen-doped coronene models but higher than those of boron-doped and pure coronene models. These results indicate that the rate of isotope permeation is higher in BN-doped coronene than that of nitrogen-doped coronene but lower than boron-doped and pure coronene models (Fig. 7).

The permeation barriers for proton isotopes are increased when 2(BN) atoms are incorporated in the central hexagon. The H^+^ and D^+^ faced similar potential barriers (1.20 eV) in 2(BN)-coronene sheet remained, while for T^+^, it is 1.21 eV, respectively. Similarly, when 3(BN) atoms are introduced into the ring, the barriers further reduced compared to 2(BN)-coronene. The potential barriers of 1.16 eV, 1.17 eV and 1.18 eV are observed for H^+^, D^+^ and T^+^, respectively. This shows that increasing number of nitrogen atoms in the ring are offering attractive interactions to the passing protons due to which the barriers are rising.

Relative selectivity ratios calculated from zero-point correction differences have shown an irregular pattern. For BN-coronene, K_H^+^_/K_D^+^_ and K_H^+^_/K_T^+^_ were calculated as 1.71 and 2.10, respectively. In case of 2(BN)-coronene, these ratios are increased to 2.08 and 2.39 for K_H^+^_/K_D^+^_ and K_H^+^_K_T^+^_ respectively. However, the selectivity ratios are lower in the case of 3(BN)-coronene where the calculated K_H^+^_/K_D^+^_ and K_H^+^_K_T^+^_ are 1.38 and 1.62, respectively.

### 3.6 Permeation of proton isotopes through boron and nitrogen (BN)-doped dodecabenzocoronene models

**Fig. 8:**
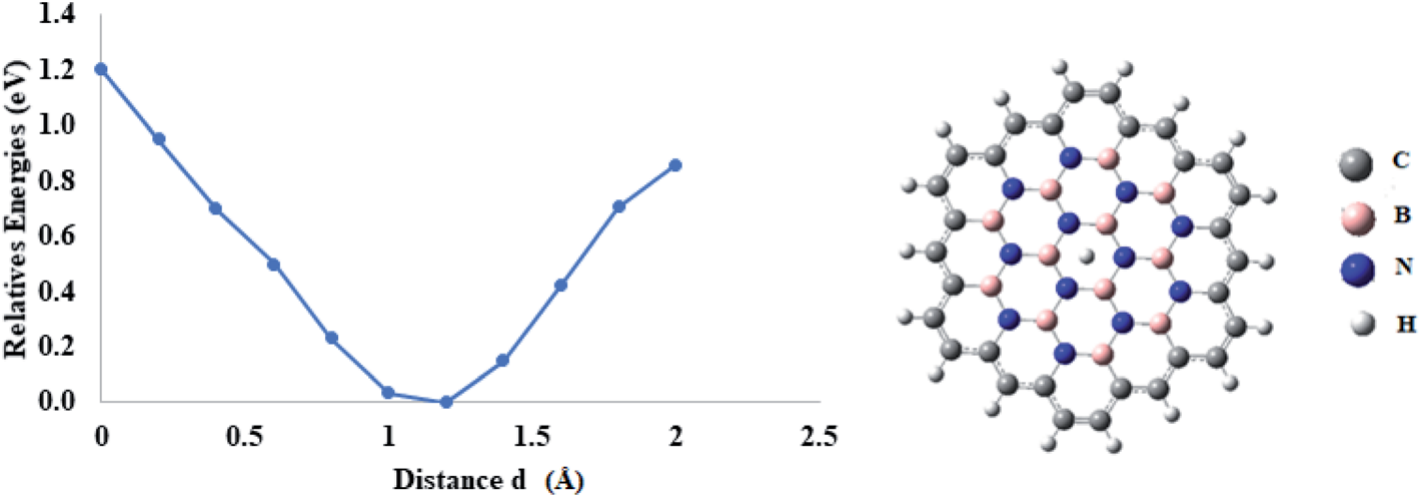
Permeation of proton isotopes through a *n*BN-doped DBC model.

It is interesting but surprising to note that coronene with a BN hexagon at the center has higher permeation barriers than those of pure h-BN, which can be attributed to the immediate environment of the carbon atom. We became interested in studying whether having a pure coronene analogue of BN in a big polycyclic aromatic compound still has this effect or not. For this purpose, dodecabenzocoronene was selected. The dodecabenzocoronene (DBC) is a stable aromatic compound and it is the extension of coronene molecule. It consists of nineteen benzene rings arranged as a hexagonal structure.^51^ Dodecabenzocoronene can easily accommodate the coronene analogue of BN in its geometry (see Fig. 8). In this model, the permeation barriers are again compared with some selected dopant numbers.

First, two carbons atoms of the inner ring of DBC were doped by two B atoms and proton isotopes were allowed to pass through the central pore. In this case, the permeation of protium H^+^ and deuterium D^+^ isotopes was studied. The energy barrier for the permeation of H^+^ and D^+^ was 0.70 eV and 0.71 eV, respectively. These barriers are comparatively higher than those calculated when two boron atoms were doped on a coronene sheet. Similarly, when boron and nitrogen atoms are doped simultaneously on the DBC, the calculated barriers for H^+^ and D^+^ are again higher than those of BN-coronene (Table 5).

**Table 5:** Permeation barriers (*E*_p_) for proton isotopes through BN co-doped coronene and DBC sheets

Model	Permeation barriers (*E*_p_) in eV		
	Protium (H^+^)	Deuterium (D^+^)	Tritium (T^+^)
BN-coronene	1.15	1.16	1.17
2(BN)-coronene	1.20	1.20	1.21
3(BN)-coronene	1.16	1.17	1.18
2B-DBC	0.70	0.71	—
BN-DBC	1.26	1.29	—
*n*BN-DBC	1.24	1.27	—

Finally, DBC is co-doped with ‘*n*’ number of BN atoms in its central core. In the case of *n*(BN) dopants, the computed energy barrier is 1.24 eV for the H^+^ isotope, while in the case of D^+^, this energy barrier is 1.27 eV. The energy barriers observed for H^+^ and D^+^ in the case of *n*BN-doped DBC models are lower than those of pure h-BN sheets (1.48 eV) as reported by Zhang *et al.*^34^ In pure h-BN, van der Waals radius is larger than that of the *n*BN-doped DBC model. The calculated radii are 1.44 and 1.41 Å for h-BN and *n*BN dodecabenzocoronene models, respectively. On the other side, the permeation rate is comparable in both cases (*n*BN-doped DBC models). Therefore, it can be safely concluded that the presence of carbon atoms on the periphery of the BN model provide additional effect for the permeation of proton isotopes. This study illustrates the need for permeation study through other hetero-2D surfaces, where interesting hidden chemistry is still unexplored.

### 3.7 Natural bond orbital and Frontier molecular orbital analysis

NBO charges were studied to find the direction of charge transfer between H^+^ and bare and doped coronene sheets. We performed electronic properties for H^+^ only, because electronic properties of D^+^ and T^+^ are identical to those of H^+^ doped systems (due to the same number of electrons). The charge on H^+^ in each case obtained through the NBO analysis is reported in Table S1.† The positive charge is noticed on H^+^ in case of pure h-BN, and nB-coronene models, but this charge is less than +1 (initial charge), which indicate that the electronic cloud is donated by h-BN sheet and nB-coronene models. In the case of B-coronene and 2B-coronene models, charges on H^+^ are 0.50 e^−^ and 0.56 e^−^ respectively. However, a decrease in the positive charge intensity is noticed as the number of B-atoms increases in the coronene model (see Table S1†). For H^+^@*n*N-coronene models, the charge on H^+^ is negative, which shows that N-doped coronene models shift the charges towards H^+^. Among studied H^+^@*n*N-coronene models, higher exchange of charges is observed in the cases of 2N-coroene (−0.69 e^−^), 3N-coronene (−0.81 e^−^) and 6N-coronene (0.60 e^−^). In the rest of the boron-nitrogen-doped coronene models, transfer of the charges is noticed from doped coronene to H^+^. In all designed systems, N-doped coronene models shift the highest amount of charge towards H^+^ compared to other doped coronene models.

The HOMO–LUMO energy gap of 6.07 eV and 7.06 eV is observed in the case of pure coronene and h-BN sheets. The HOMO–LUMO gaps for all the doped coronene models are given in Table S1.† In the case of B-doped coronene models, the decrease in HOMO–LUMO gaps is more prominent as the number of boron atoms increases in the coronene sheet. Among the studied model of B-doped coronene, the highest decrease in the HOMO–LUMO gap is observed in the case of 6B-coronene (3.48 eV). The appreciable change in HOMO–LUMO energy gaps of B-doped coronene models occurred due to the increase and decrease in energies of HOMOs and LUMOs, respectively. In the case of N-doped coronene model carrying H^+^, there is irregular change in HOMO–LUMO gaps with the increase in N-atoms. However, a significant decrease in HOMO–LUMO gaps is observed in the case of 2N-coronene (3.10 eV) and 3N-coroene (3.23 eV) compared to pure coronene sheets (6.07 eV). The increase in the conductivity of H^+^@BN-coronene and H^+^@BN-DBC models is attributed to more increase and decrease in energies of their HOMOs and LUMOs, respectively.

## 4 Conclusions

The permeation of proton isotopes H^+^, D^+^ and T^+^ has been studied through boron- and nitrogen-doped graphene membranes. The permeation barriers of undoped graphenes (coronene model) are higher than those of boron-doped analogues. This is because the van der Waals radii are greater in undoped coronene models, which decreases the pore size for proton permeation. However, boron-doped coronene models have smaller van der Waals radii, which increase the pore size for proton permeation. Moreover, by increasing the number of boron dopants from 1 to 6 atoms, a decrease in permeation barrier was observed. For boron doping, the highest energy barrier (∼0.77 eV) is observed in mono-boron-doped coronene models, whereas the lowest energy barrier (∼0.04 eV) was observed in hexa-boron-doped coronene models. Therefore, the permeation of proton is faster in hexa-boron-doped coronene models. In p-type materials, the formation of holes is also a reason for the fast permeation of proton isotopes. An odd-even pattern of ZPE was obtained by nitrogen doping for proton permeation through coronene models due to the unfavorable radical effect of doped nitrogen atoms. The observed order of permeation rate is 1 < 2 < 4 < 3 < 6 < 5 in nitrogen-doped models. BN co-doped models also show an odd-even pattern in ZPE. The order of permeation rate is 1 < 3 < 2 in BN-doped models because of unfavorable interactions of nitrogen atoms with proton isotopes. Furthermore, we studied the isotope permeation through higher analogue DBC. Most of the trends are similar to those of the coronene model; however, the selectivity reversal is also observed in *n*BN- and 2(BN)-doped DBC models due to unfavorable interactions of nitrogen atoms. The permeation rate of proton isotopes is lower in DBC models than that of coronene models due to greater interactions in DBC models. Our studies suggest that boron-doped graphene models show a very low energy barrier of ∼0.04 eV for the permeation of proton compared to the pure graphene model and h-BN sheets, while nitrogen-doped graphenes have a very high energy barrier up to 7.44 eV for permeation. Therefore, boron-doped graphene models are suitable candidates for proton permeation. Moreover, the presence of carbon atoms in the periphery of BN sheets has significant negative effects on the permeation of proton isotopes, an unexplored dimension of remote neighboring effect in 2-D materials. This study illustrates the need for permeation study through other hetero-2D surfaces, where interesting hidden chemistry is still unexplored.

## Conflicts of interest

There are no conflicts to declare.

## Supplementary Material

RA-012-D1RA09398C-s001
