# Supplementary material for: Permeability of boron- and nitrogen-doped graphene nanoflakes for protium/deuterium ions
Source: RSC Adv. 2022 Jan 31;12(7):3883–91. doi: 10.1039/d1ra09398c (PMC8981073; doi:10.1039/d1ra09398c)
Supplement: RA-012-D1RA09398C-s001 [file RA-012-D1RA09398C-s001.pdf]

Electronic Supplementary Material (ESI) for RSC Advances.
This journal is © The Royal Society of Chemistry 2022

Supporting Information

Permeability of Boron and Nitrogen Doped Graphene Nanoflakes for Protium/Deuterium Ions

Iram Gul[a], Muhammad Yar[a], Arsalan Ahmed[a], Muhammad Ali Hashmi[b], Khurshid Ayub[a]

[a] Department of Chemistry, COMSATS University, Abbottabad Campus, Abbottabad, KPK, Pakistan 22060

[b] Department of Chemistry, University of Educations, Attock Campus, Attock, Punjab, Pakistan 43600

Correspondence

Phone: +92-992-383591-6

Fax: +92-992-383441

khurshid@cuiatd.edu.pk

Table S1. Electronic properties of pure, H^+@N-coronene , H^+@nB-coronene and formation energies of each surface.

H^+ permeability through pure and doped (B,N) coronene models	Charge on H^+	HOMO	LUMO	E_{H-L}(eV)	Formation energy per atom (kcal/mol)
Coronene	0.58	-11.05	-4.98	6.07	-160.14
Pure *h*-BN	0.54	-12.81	-5.75	7.06	-142.43
B-Coronene	0.50	-11.03	-4.13	6.90	-157.02
2B-Coronene	0.56	-10.90	-6.68	4.22	-153.61
3B-Coronene	0.45	-11.35	-6.56	4.80	-150.38
4B-Coronene	0.37	-10.54	-6.76	3.78	-146.48
5B-Coronene	0.33	-10.92	-5.98	4.93	-142.69
6B-Coronene	0.31	-10.35	-6.87	3.48	-137.91
N-Coronene	-0.23	-11.08	-4.47	6.61	-158.13
2N-Coronene	-0.69	-8.24	-5.14	3.10	-155.91
3N-Coronene	-0.81	-8.32	-5.09	3.23	-153.75
4N-Coronene	-0.40	-9.12	-5.29	3.83	-150.57
5N-Coronene	-0.46	-9.24	-4.20	5.04	-147.45
6N-Coronene	-0.60	-8.92	-4.85	4.07	-144.24
BN-Coronene	0.52	-10.76	-5.07	5.68	-157.20
2(BN)-coronene	0.48	-10.51	-5.23	5.28	-155.06
3(BN)-Coronene	0.53	-10.50	-5.73	4.78	-153.64
2B-DBC	0.47	-9.24	-4.95	4.28	-169.99
2B2N-DBC	0.48	9.43	-4.81	4.63	-162.79
nBN-DBC	-0.13	-8.83	-6.28	2.54	-170.71
